# Far-Infrared-Emitting Sericite Board Upregulates Endothelial Nitric Oxide Synthase Activity through Increasing Biosynthesis of Tetrahydrobiopterin in Endothelial Cells

**DOI:** 10.1155/2019/1813282

**Published:** 2019-10-31

**Authors:** Seonhee Kim, Ikjun Lee, Hee-Jung Song, Su-jeong Choi, Harsha Nagar, Sung-min Kim, Byeong Hwa Jeon, Book Sung Kim, Hyun Jong Park, Shuyu Piao, Cuk-Seong Kim

**Affiliations:** ^1^Department of Physiology & Medical Science, School of Medicine, Chungnam National University, Daejeon 301-747, Republic of Korea; ^2^Department of Neurology, Chungnam National University Hospital, School of Medicine, Chungnam National University, Daejeon 301-747, Republic of Korea; ^3^Korea Textile Development Institute, Daegu 418-42, Republic of Korea; ^4^Gumcheon Corporation, Okcheon-gun, Chungcheongbuk-do 373-801, Republic of Korea

## Abstract

Far-infrared ray (FIR) therapy has been reported to exert beneficial effects on cardiovascular function by elevating endothelial nitric oxide synthesis (eNOS) activity and nitric oxide (NO) production. Tetrahydrobiopterin (BH_4_) is a key determinant of eNOS-dependent NO synthesis in vascular endothelial cells. However, whether BH_4_ synthesis is associated with the effects of FIR on eNOS/NO production has not yet been investigated. In this study, we investigated the effects of FIR on BH_4_-dependent eNOS/NO production and vascular function. We used FIR-emitting sericite boards as an experimental material and placed human umbilical vein endothelial cells (HUVECs) and Sprague–Dawley rats on the boards with or without FIR irradiation and then evaluated vascular relaxation by detecting NO generation, BH_4_ synthesis, and Akt/eNOS activation. Our results showed that FIR radiation significantly enhanced Akt/eNOS phosphorylation and NO production in human endothelial cells and aorta tissues. FIR can also induce BH_4_ storage by elevating levels of enzymes (e.g., guanosine triphosphate cyclohydrolase-1, 6-pyruvoyl tetrahydrobiopterin synthase, sepiapterin reductase, and dihydrofolate reductase), which ultimately results in NO production. These results indicate that FIR upregulated eNOS-dependent NO generation via BH_4_ synthesis and Akt phosphorylation, which contributes to the regulation of vascular function. This might develop potential clinical application of FIR to treat vascular diseases by augmenting the BH_4_/NO pathway.

## 1. Introduction

Vascular endothelial cells, which are located in the inner surfaces of blood vessels, have a pivotal role in modulating vascular tone and blood fluidity through the release of vasoactive substances [1]. The endothelial isoform of nitric oxide synthase (eNOS) is robustly expressed in endothelial cells, and is a main source of vascular nitric oxide (NO), a major endothelium-dependent relaxing factor [2]. Tetrahydrobiopterin (BH_4_) is a key regulator of eNOS in the setting of cardiovascular diseases, and augmenting BH_4_ bioavailability within the endothelium enhances NO production and therefore prevent the development of cardiovascular diseases [3]. BH_4_ is synthesized by two pathways: the de novo pathway and the recycling pathway. Guanosine triphosphate cyclohydrolase-1 (GCH1), the rate-limiting enzyme in BH_4_ biosynthesis, is the main determinant of BH_4_ levels [4]. Dihydrofolate reductase (DHFR), an enzyme that recycles oxidized BH_4_, can also catalyze the formation of 7,8-dihydrobiopterin (BH_2_) to BH_4_, resulting in eNOS-dependent NO production. [3] Additionally, evidence has shown that Akt, a downstream target of phosphatidylinositol 3-kinase (PI3K), also activates eNOS phosphorylation in endothelial cells [5]. Therefore, both the PI3K/Akt pathway and BH_4_ synthesis play a role in regulating blood vessel vasodilatation via eNOS/NO production.

Far-infrared ray (FIR) radiation consists of invisible electromagnetic waves with wavelengths between 3 and 1000 *μ*m, longer than those of visible light [6]. FIR energy in the form of heat is perceived in surrounding tissues by thermoreceptors in the skin [7]. Emerging studies have indicated that FIR therapy improves the health of patients with cardiovascular diseases, diabetes, inflammation, and other diseases [8–11]. However, these studies revealed only that FIR therapy has a protective effect on impaired vascular diseases; the underlying molecular mechanisms of the effects of FIR on eNOS/NO production have not been investigated. In this study, we investigated the whether BH_4_ synthesis and Akt phosphorylation are associated with the effects of FIR-emitting sericite boards on vasodilation via eNOS/NO production.

## 2. Materials and Methods

### 2.1. Cell Culture

Human umbilical vein endothelial cells (HUVECs) were obtained from Clonetics (San Diego, CA, USA), and all cells were cultured in endothelial growth medium 2 from Lonza (Walkersville, MD, USA) at 37°C with 5% CO_2_. Cells between passages 2 and 8 were used for all experiments. HUVECs grown to 80% confluence were incubated with or without FIR-emitting sericite boards for different time points. In tumor necrosis factor (TNF)-*α* treatment experiments, HUVECs grown to 70% confluence were incubated with or without FIR-emitting sericite boards for 24 h and then stimulated with 10 ng/ml human recombinant TNF-*α* (Sigma Aldrich, Cat. No. T0157) for another 24 h.

### 2.2. FIR-Emitting Sericite Boards

The sericite boards were produced with both polyethylene terephthalate virgin chips (0.6 intrinsic viscosity) and sericite nanoparticles (mean diameter 300 *μ*m; Gumcheon Corporation, Korea) manufactured by the Korea Textile Development Institute (Daegu, Korea). The FIR-emitting sericite boards used in this study have a length of 20.5 cm, a width of 16.5 cm, and a thickness of 0.2 cm. The content of sericite in the masterbatch was approximately 20% by weight. The spectra of the FIR-emitting sericite wooden boards exhibited high emissivity values (>0.8) in a wavelength range of 5∼20 *μ*m as measured by the Korea Textile Development Institute.

### 2.3. Animal Model

All animal studies were performed in the animal facility following the guidelines of the Institutional Animal Use and Care Committee at Chungnam National University. All experiments were approved by the institutional review board of Chungnam National University (No. CNUH-017-A0013). Nine-week-old male Sprague–Dawley (SD) rats (Samtako, Osan, Korea) were housed in a controlled environment (12-h day/night cycle; humidity 50 ± 60%; temperature 23°C) and fed chow (Harlan, Indianapolis, IN, USA) *ad libitum* and autoclaved water. The rats were randomly divided into two groups (*n* = 5 per group). The control group comprised rats kept on a sericite board without FIR radiation for 3 or 7 days, and the FIR treatment group comprised rats kept on an FIR-emitting sericite board for 3 or 7 days. At the experimental endpoint, all animals were anesthetized and euthanized using urethane (1.2 g/kg, i.p.). After taking blood by heart puncture, a midsternal split was quickly performed, and the descending thoracic aorta was carefully excised and used for experiments.

### 2.4. Nitrite and Nitrate Measurements

The NO metabolites nitrite and nitrate, stable breakdown products of NO, were quantified using the Nitric Oxide Assay Kit (Colorimetric) (Cat. No. ab65328; Abcam, Cambridge, UK) according to the manufacturer's instructions. The phenol red-free medium used for the incubation of HUVECs or rat plasma was deproteinized using a 10-kDa cutoff filter (Microcon YM10 Millipore, Burlington, MA, USA). After subtraction of background fluorescence, values were normalized to obtain the total amount of protein.

### 2.5. Antibodies and Immunoblotting

The following antibodies were used: anti-Akt (Cell Signaling Technology, Danvers, MA, USA), antiphospho Akt (Santa Cruz Biotechnology, Santa Cruz, CA, USA), anti-*β*-actin (Sigma-Aldrich, St. Louis, MO, USA), anti-eNOS (Santa Cruz Biotechnology), antiphospho eNOS (S1177) (Cell Signaling Technology), antivascular cell adhesion molecule (VCAM)-1 (Santa Cruz Biotechnology), anti-intercellular adhesion molecule (ICAM)-1 (Santa Cruz Biotechnology), anti-GCH1 (Santa Cruz Biotechnology), anti-6-pyruvoyl tetrahydrobiopterin synthase (PTS) (Santa Cruz Biotechnology), antisepiapterin reductase (SPR) (Santa Cruz Biotechnology), and anti-DHFR (Santa Cruz Biotechnology). Immunoblotting was performed as previously described [12].

### 2.6. High-Performance Liquid Chromatography (HPLC) Sample Preparation

HUVECs and rat lung endothelial cells were used to measure the biopterin content. To harvest rat lung endothelial cells, we followed methods described in a previous paper [12, 13]. Cells were dissolved in cell lysis buffer (50 mM Tris-HCl, pH 7.5, 150 mM NaCl, 1 mM EDTA, and 1 mM DTT) and centrifuged at 13000 g for 20 min. After centrifugation, protein extraction buffer (1 : 1 mixture of 1.5 M HClO_4_ and 2 M H_3_PO_4_) was added at 10% of the sample volume, followed by incubation for 5 min. The samples were then centrifuged, and protein-free supernatants were collected.

### 2.7. Measurement of Biopterin

Fluorometric HPLC analysis was used to measure biopterins. Measurement of BH_4_ was performed by a differential oxidation method as previously described [14]. Briefly, different oxidation methods (acid oxidation for total biopterin and alkali oxidation for BH_2_ and biopterin) were used. Iodine solution (1% iodine in 2% KI solution) was added into sample (10% of final volume), and 1 M NaOH was added to alkali oxidation sample additionally. Samples were incubated at room temperature for 1 h in the dark. Then, 20% of 1 M H_3_PO_4_ was added to alkali oxidation samples, and 5% ascorbic acid (20 mg/ml) was added to all samples. Detection was performed using 1290 series fluorescence detector (Agilent Technologies, Santa Clara, CA, USA; 350 nm excitation, 450 nm emission) and 120 SB-C18 column (Poroshell) with mobile phase (5 : 95 MeOH-water, v/v, 0.5 ml/min), and all protocols were performed at the Korea Basic Science Institute (Seoul, South Korea). BH_4_ amounts were calculated by subtracting BH_2_ + biopterin from total biopterin. Biopterin content was expressed as relative to the control group.

### 2.8. Vascular Reactivity

After cleaning the excess fat, the isolated rat aortas were placed in ice-cold Krebs buffer (118.3 mM NaCl, 4.7 mM KCl, 2.5 mM CaCl_2_, 1.2 mM KH_2_PO_4_, 25 mM NaHCO_3_, 1.2 mM MgSO_4_, 11 mM glucose, and 0.0026 mM CaNa_2_ EDTA). The aortas were cut transversely into 5–10 rings (2.0–3.0 mm) and maintained with Krebs buffer solution at 37°C and pH 7.4. Endothelium-dependent vasorelaxation from SD rats was measured in aortic rings as previously described [15].

### 2.9. Quantitative Real-Time Polymerase Chain Reaction

Total RNA from HUVECs or lung endothelial cells were isolated using a protocol described previously. [12] The primers used for human VCAM-1 and ICAM-1 were as follows: sense VCAM-1, 5′-AAT GGG AAT CTA CAG CAC CTT T-3′ and antisense VCAM-1, 5′-ATA TCC GTA TCC TCC AAA AAC T-3′; sense ICAM-1, 5′-GTG GTA GCA GCC GCA GTC-3′ and antisense ICAM-1, 5′-GGC TTG TGT GTT CGG TTT CA-3′. The primers for human glyceraldehyde 3-phosphate dehydrogenase, amplified as an internal control, were as follows: sense, 5′-CCT GCA CCA CCA ACT GCT TA-3′ and antisense, 5′-GGC CAT CCA CAG TCT TCT GAG-3′. The relative quantitation of each mRNA was calculated using 2^−ΔΔCt^ and was expressed as mean ± standard error of the mean (SEM).

### 2.10. Statistical Analysis

Statistical analysis was performed using SPSS statistical software (version 17.0; SPSS Inc., Chicago, IL, USA). Differences between groups were compared using unpaired *t*-tests. For multiple comparisons, one-way analysis of variance (ANOVA) was performed by Tukey's post hoc tests. Data are presented as the mean ± standard error of mean (SEM). *P* values less than 0.05 were considered statistically significant.

## 3. Results

### 3.1. FIR Radiation Stimulated Akt/eNOS Phosphorylation and NO Production in HUVECs

**Figure 1:**
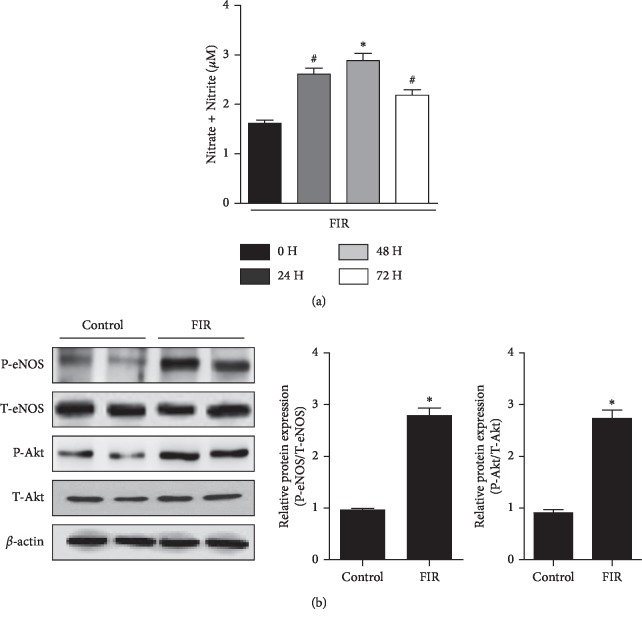
FIR radiation stimulates Akt/eNOS phosphorylation and NO production in HUVECs. (a) Cellular NO levels were measured with FIR emitting sericite board for 0, 24, 48, and 72 hours. (b) Phosphorylation of Akt and eNOS was determined after 48 hours of cultivation with FIR radiation. *β*-actin is shown as a loading control. Data are shown as the mean ± SEM of three independent experiments. ^*∗*^*P* < 0.01 compared with control cells. ^#^*P* < 0.05 compared with control cells.

NO is a major endothelium-derived relaxing factor that plays a vital role in the regulation of vascular tone and function [16]. Extensive literature indicates that phosphorylation by Akt on Ser1179 of eNOS leads to augmentation of NO production [5, 17]. In order to investigate the effect of FIR-emitting sericite boards on NO production in endothelial cells, we placed the boards under the plate of HUVECs and incubated for different time points. Maximal increase in NO production was observed after 48 h incubation (*P*=0.008) (Figure 1(a)). Thus, we first assessed whether FIR radiation induced Akt/eNOS activation in 48 h incubation in HUVECs. As shown in Figure 1(b), eNOS and Akt phosphorylation in HUVECs was significantly induced in the FIR radiation group compared with the non-FIR radiation group (*P*=0.004 and *P*=0.008, respectively). These results indicate that FIR radiation stimulates the production of endothelial NO via an Akt/eNOS signaling pathway in HUVECs.

### 3.2. FIR Radiation Induced BH_4_ Synthesis in HUVECs

**Figure 2:**
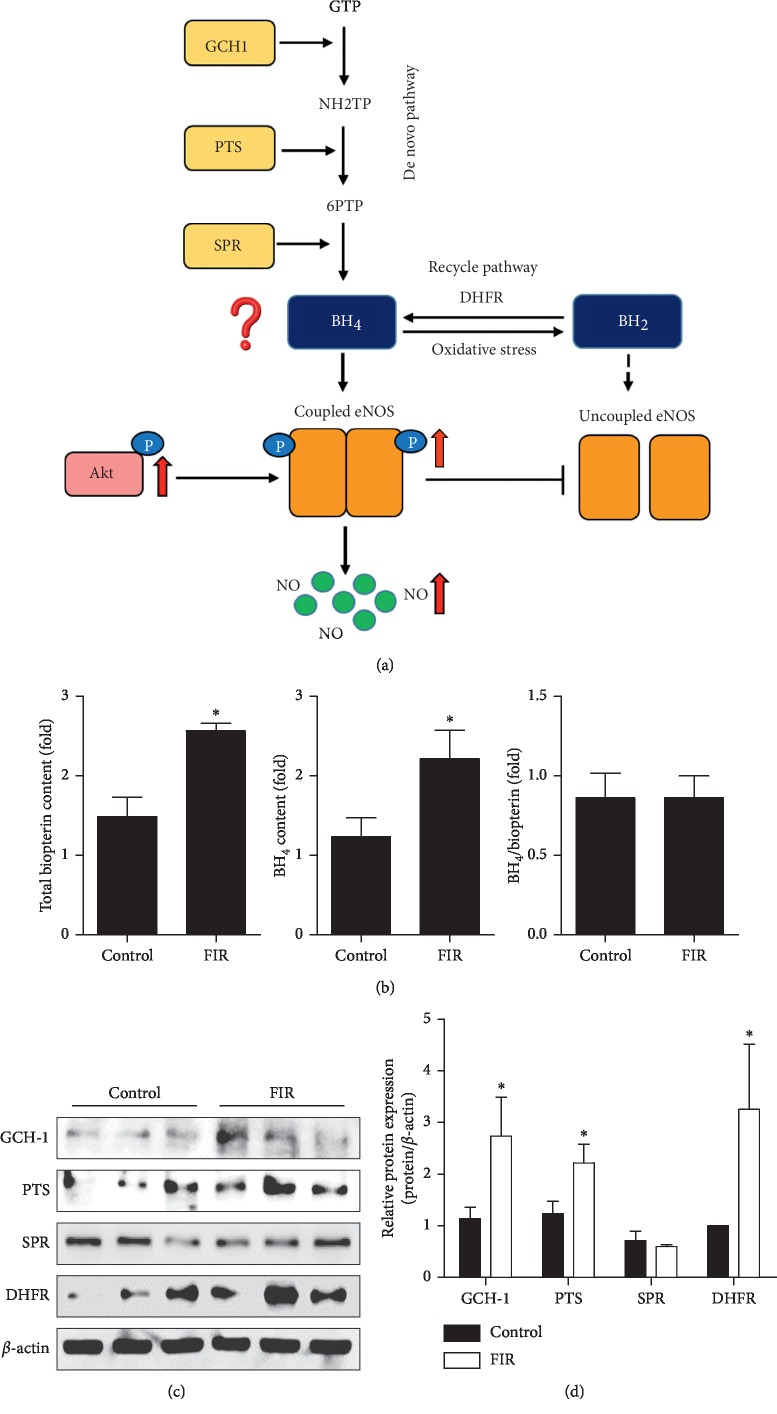
FIR radiation induces BH_4_ synthesis in HUVECs. HUVECs were cultured with or without FIR-emitting sericite board for 48 hours. (a) Schematic model summarizing the mechanism of FIR-induced improvement of vascular function. (b) Intracellular levels of total biopterin and BH_4_. (c) The protein expression of GCH1, PTS, SPR, and DHFR. *β*-actin is shown as a loading control. (d) Protein levels were quantified by densitometric analysis. Data are shown as the mean ± SEM of three independent experiments. ^*∗*^*P* < 0.05 compared with control cells.

BH_4_ is a key cofactor for the synthesis of NO by eNOS catalysis and plays an important role in regulating the balance between NO and superoxide production (Figure 2(a)). We further explored whether the increased eNOS activation was regulated by BH_4_ biosynthesis.  Figure 2(b) showed that total biopterin and intracellular BH_4_ levels were elevated (*P*=0.026 and *P*=0.009, respectively) along with no change in the BH_4_/biopterin ratio (*P*=0.390). After incubation of cells on FIR-emitting sericite boards for 48 h, the protein expression of GCH1, PTS, and DHFR was enhanced more than two-fold compared with the control group (*P*=0.049, *P*=0.043, and *P*=0.046, respectively) (Figures 2(c) and 2(d)), indicating that both synthesis pathways were induced. Collectively, these data show that FIR radiation had an effect on the maintenance of eNOS coupling by regulating steady-state BH_4_ levels.

### 3.3. FIR Radiation Stimulated Akt/eNOS Phosphorylation and NO Production *In Vivo*

**Figure 3:**
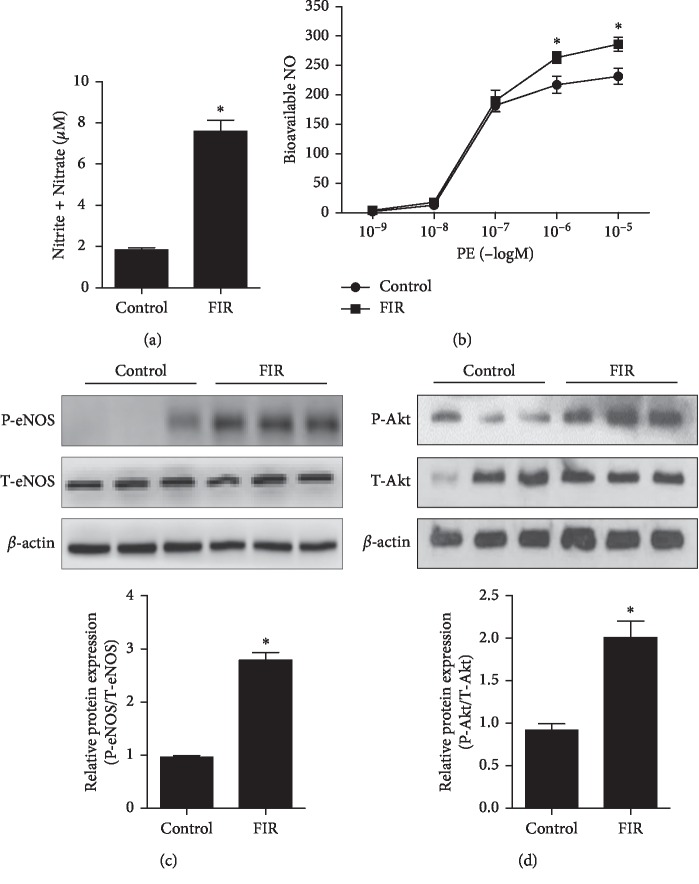
FIR radiation stimulates Akt/eNOS phosphorylation and NO production in vivo. Rats were exposed with or without FIR-emitting sericite board for 7 days. (a) NO production level was measured in the plasma of SD rats. (b) Concentration-response curves to phenylephrine in isolated aortic rings. (c) Phosphorylation of eNOS. (d) Phosphorylation of Akt protein levels from the aortic rings. *β*-actin is shown as a loading control. Data are shown as the mean ± SEM of three independent experiments. ^*∗*^*P* < 0.05 compared with control cells.

**Figure 4:**
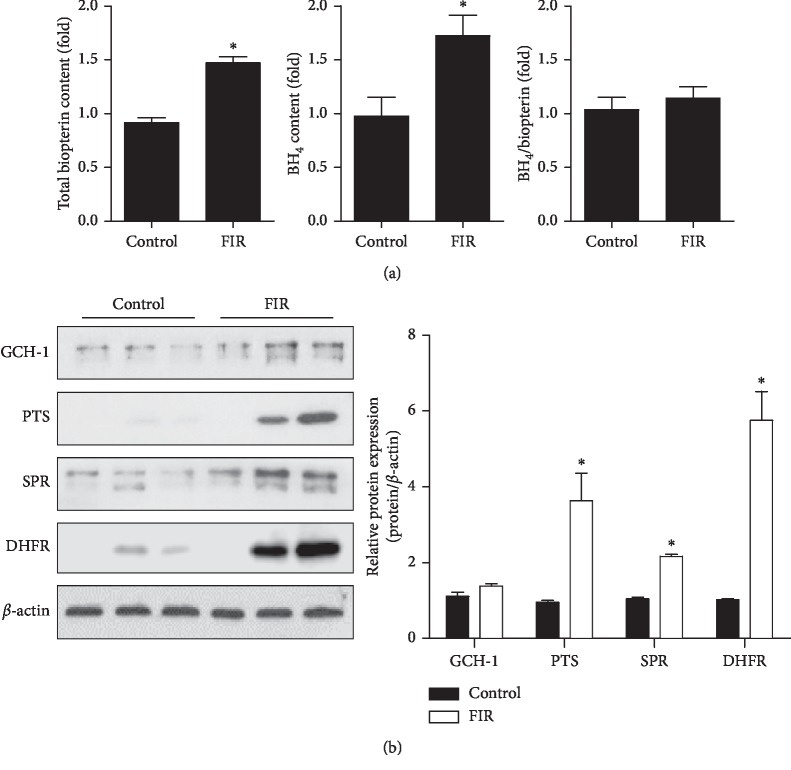
FIR radiation induces tetrahydrobiopterin (BH_4_) synthesis in vivo. Rats were exposed with or without FIR-emitting sericite board for 7 days. (a) Lung endothelial cells were isolated from control and FIR group rats using CD31 and CD45 beads. Endothelial biopterin and BH_4_ levels were quantified by HPLC analysis. (b) GCH1, PTS, SPR, and DHFR protein expression in aorta tissues. *β*-actin is shown as a loading control. Protein levels were quantified by densitometric analysis. Data are shown as the mean ± SEM of three independent experiments. ^*∗*^*P* < 0.05 compared with control cells.

To further observe the effect of FIR-emitting sericite boards on Akt/eNOS/NO pathway *in vivo,* we exposed rats to FIR-emitting sericite boards for 3 or 7 days. Western blot results indicated that aortic eNOS phosphorylation on day 7 showed higher expression compared to control group (supplementary [Supplementary-material supplementary-material-1]). We next examined the plasma levels of NO and vascular relaxation in the rat thoracic aorta at the end of 7 days of experiment. As shown in Figure 3(a), elevated NO production was observed in the FIR radiation group (an approximately 4-fold increase, *P*=0.012) compared with the control group. Furthermore, we investigated the effects of FIR on phenylephrine-induced vasorelaxation in the aortic rings. FIR treatment induced an increase in the concentration-dependent relaxation of isolated aortic rings (Figure 4(b)), which were precontracted with phenylephrine. In addition, both eNOS and Akt phosphorylation were significantly enhanced in the FIR treatment group (*P*=0.015 and *P*=0.034, respectively), which was consistent with the results observed in HUVECs (Figures 3(c) and 3(d)). These results collectively suggest that vascular function, as well as Akt/eNOS activity, is improved with FIR radiation of vascular endothelial cells *in vivo*.

### 3.4. FIR Radiation Induced BH_4_ Synthesis *In Vivo*

Our previous results showed that FIR radiation induced BH_4_ synthesis in HUVECs by promoting de novo and recycling pathways (Figure 2). To determinate whether FIR radiation affects BH_4_ synthesis *in vivo*, we reared rats with or without FIR radiation for 7 days and then purified CD31+/CD45-endothelial cells from lung tissue for BH_4_ measurement by HPLC measurement. As expected, the FIR radiation group expressed higher total biopterin and BH_4_ levels compared with the control group (*P*=0.001 and *P*=0.023, respectively), but no change in the BH_4_/biopterin ratio (*P*=0.270) (Figure 4(a)). We next determined the protein expression (GCH1, PTS, SPR, and DHFR) in aortic tissues. Figure 4(b) showed that the FIR treatment group had significantly increased expression of PTS, SPR, and DHFR (*P*=0.044, *P*=0.045, and *P*=0.045, respectively), but not GCH-1 (*P*=0.200). These results suggest that FIR radiation induced BH_4_ synthesis for NO production *in vivo.*

## 4. Discussion

With the development of new technologies, delivering FIR radiation has become safe and effective, and is now widely used to promote health [6]. Many studies have demonstrated the biological effects of FIR devices on vascular function [18, 19]. However, compared to FIR devices and saunas, FIR-emitting materials are more easily applied for different medical conditions [20, 21]. The FIR-emitting sericite boards used in this study were made from sericite nanoparticles, which could be incorporated into fibers and woven into fabrics for textile manufacture. In the present study, we investigated whether FIR-emitting sericite boards had a protective effect on vascular function and revealed the molecular mechanisms underlying this effect. We demonstrated that FIR-emitting boards promoted vascular relaxation by stimulating the eNOS/NO pathway via Akt activation and BH_4_ synthesis.

Endothelial basal NO production catalyzed by eNOS is a fundamental determinant of vascular homeostasis, regulating angiogenesis and blood pressure. Decreased NO levels impair endothelium-dependent vasorelaxation and eventually lead to hypertension [22]. Several studies have shown that FIR radiation therapy increased NO production through eNOS phosphorylation in different endothelial cells [23, 24]. Consistent with these studies, upregulated p-eNOS/NO expression was observed in FIR radiation-treated endothelial cells compared with control cells (Figures 1 and 3). In addition, our previous studies reported that Akt is involved in eNOS activation and induced NO production under different experimental conditions [25, 26]. In the present study, we found that FIR treatment significantly stimulated Akt/eNOS phosphorylation (Figures 1 and 3), which may be one of the upstream pathways for NO production.

BH_4_ is another essential cofactor for eNOS activity that regulates the balance between NO and ROS via coupling and uncoupling eNOS, which is synthesized by de novo and recycling pathways from GTP and BH_2_, respectively [27]. BH_4_ levels are synthesized through the production of rate-limiting enzymes involving GCH1, PTS, and SPR [28]. In addition, DHFR-mediated recycling pathway can determine cellular BH_4_ homeostasis by reductase BH_2_ activity [29]. A recent work by Jade Bailey et al. reported that BH_4_ regulated not only NO production but also ROS expression via eNOS [30], indicating that a BH_4_ deficiency in endothelial cells results in decreased NO and increased ROS generation via eNOS uncoupling. Because the elevated production of eNOS-derived NO was observed in cells exposed to FIR radiation (Figure 1), we specifically tested whether BH_4_ synthesis and DHFR, GCH1, PTS, and SPR expression were increased. Indeed, we found that the elevated enzymes (DHFR, GCH1, and PTS) in the FIR radiation group augmented endothelial levels of BH_4_ (Figure 2), and that the maintenance of intracellular BH_4_ homeostasis contributes to inducing eNOS coupling and ultimately NO production. Our studies suggest that FIR radiation therapy upregulated eNOS/NO expression by increasing Akt activation and BH_4_ levels in HUVECs. Long term of FIR-irradiated wooden board has significant antihypertensive effects in hypertensive rats from 1 week [31]. To further explore the protective effects of FIR radiation on vascular function in an animal model, we used 9-week-old SD rats to study the efficacy of FIR radiation for 3 or 7 days. Similar with the study, rats raised in FIR-emitting sericite boards for 7 days significantly activated Akt/eNOS/NO expression and increased BH_4_ levels compared with the rats in the control group (Figures 3 and 4). However, different from the increase of GCH1 expression, the levels of SPR in the FIR group mice showed higher expression compared with control group mice. In our study, the upregulation of DHFR by FIR has been observed in both HUVECs and rat studies, which may decrease endothelial ROS production derived from uncoupled eNOS.

An enhanced ROS generation exacerbates the development and maintenance of inflammation in vascular diseases. Adhesion molecules, such as ICAM-1 and VCAM-1, are released at early stages of vascular disease and affect leukocyte adhesion to endothelial cells, a sign of inflammation. Although our results demonstrated a potential role of FIR radiation in enhancing eNOS-/NO-related pathways, we were also curious as to whether FIR-emitting sericite boards exert potent anti-inflammatory effects. The inflammatory cytokine TNF-*α* induced vascular endothelial inflammation by increasing ICAM-1 and VCAM-1 expression in HUVECs, and incubating cells with FIR for 48 h significantly inhibited subsequent TNF-*α*-induced ICAM-1 and VCAM-1 expression (Supplementary [Supplementary-material supplementary-material-1]). These results suggest that FIR radiation exerts potent anti-inflammatory effects by inhibiting the expression of the cytokine-mediated adhesion molecules.

## 5. Conclusion

In conclusion, FIR-emitting sericite boards induce eNOS/NO upregulation via BH_4_ synthesis and Akt activation and also exerts potent antiinflammatory effects in vascular endothelial cells, which might develop potential clinical application of FIR to treat vascular diseases.

## Data Availability

The data used to support the findings of this study are available from the corresponding author upon request.

## Supplementary Materials

Supplementary Materials Supplementary Figure 1. FIR radiation stimulates eNOS phosphorylation for 3 or 7 days in vivo. Rats were exposed with or without FIR-emitting sericite board for 3 or 7 days. (A) Phosphorylation of eNOS protein levels from the aortic rings. *β*-actin is shown as a loading control. (B) p-eNOS levels were quantified by densitometric analysis. Data are shown as the mean ± SEM of three independent experiments. ^*∗*^*P* < 0.05 compared with control cells. Supplementary Figure 2. FIR radiation suppresses TNF-*α*-induced expression of VCAM-1 and ICAM-1 in HUVECs. HUVECs were incubated with or without FIR-emitting sericite board radiation for 24 h, and then treated with TNF-*α* (10 ng/ml) for another 24 h. (a) Protein levels of VCAM-1 and (b) ICAM-1 were determined by Western blot analysis. *β*-actin is shown as a loading control. (c) The mRNA levels of VCAM-1 and (d) ICAM-1 were determined by qPCR. Data are shown as the mean ± SEM of three independent experiments. ^*∗*^*P* < 0.05 compared with control cells. ^#^*P* < 0.05 compared with control cells with TNF-*α* treatment.
