# Supplementary material for: Far-Infrared-Emitting Sericite Board Upregulates Endothelial Nitric Oxide Synthase Activity through Increasing Biosynthesis of Tetrahydrobiopterin in Endothelial Cells
Source: Evid Based Complement Alternat Med. 2019 Oct 31;2019:1813282. doi: 10.1155/2019/1813282 (PMC6875339; doi:10.1155/2019/1813282)
Supplement: Supplementary Materials — Supplementary Figure 1. FIR radiation stimulates eNOS phosphorylation for 3 or 7 days in vivo. Rats were exposed with or without FIR-emitting sericite board for 3 or 7 days. (A) Phosphorylation of eNOS protein levels from the aortic rings. β-actin is shown as a loading control. (B) p-eNOS levels were quantified by densitometric analysis. Data are shown as the mean ± SEM of three independent experiments. ∗P < 0.05 compared with control cells. Supplementary Figure 2. FIR radiation suppresses TNF-α-induced expression of VCAM-1 and ICAM-1 in HUVECs. HUVECs were incubated with or without FIR-emitting sericite board radiation for 24 h, and then treated with TNF-α (10 ng/ml) for another 24 h. (a) Protein levels of VCAM-1 and (b) ICAM-1 were determined by Western blot analysis. β-actin is shown as a loading control. (c) The mRNA levels of VCAM-1 and (d) ICAM-1 were determined by qPCR. Data are shown as the mean ± SEM of three independent experiments. ∗P < 0.05 compared with control cells. #P < 0.05 compared with control cells with TNF-α treatment. [file 1813282.f1.docx]

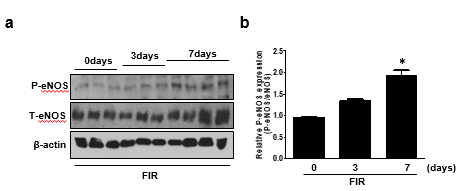


**Supplementary Figure 1. FIR radiation stimulates eNOS phosphorylation for 3 or 7 days in vivo.** Rats were exposed with or without FIR emitting sericite board for 3 or 7 days. (A) Phosphorylation of eNOS protein levels from the aortic rings. β-actin is shown as a loading control. (B) p-eNOS levels were quantified by densitometric analysis. Data are shown as the mean±SEM of three independent experiments. **P*<0.05 compared with control cells.


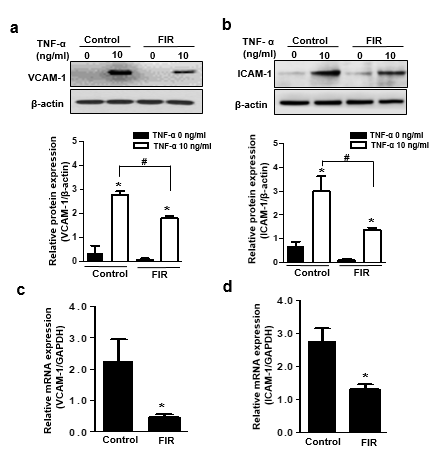


**Supplementary Figure 2. FIR radiation suppresses TNF-α-induced expression of VCAM-1 and ICAM-1 in HUVECs.** HUVECs were incubated with or without FIR emitting sericite board radiation for 24 h, then treated with TNF-α (10 ng/ml) for another 24 h. (a) Protein levels of VCAM-1 and (b) ICAM-1 were determined by western blot analysis. β-actin is shown as a loading control. (c) The mRNA levels of VCAM-1 and (d) ICAM-1 were determined by qPCR. Data are shown as the mean±SEM of three independent experiments. **P*<0.05 compared with control cells. ^#^*P*<0.05 compared with control cells with TNF-α treatment.
